# Congenital Muscle Hernia of the Leg: A Rare Case in an Infant

**DOI:** 10.5334/jbsr.2061

**Published:** 2020-04-08

**Authors:** Anne-Fleur La, Mladen Milicevic

**Affiliations:** 1 CHU Liège, BE

**Keywords:** muscle hernia, tibialis anterior, leg mass, pediatric

## Abstract

Muscle hernia is a dynamic protrusion of muscle fibers into subcutaneous tissues caused by a focal defect in the fascial sheath, rarely seen in an infant.

## Case History

**Figure 1:**
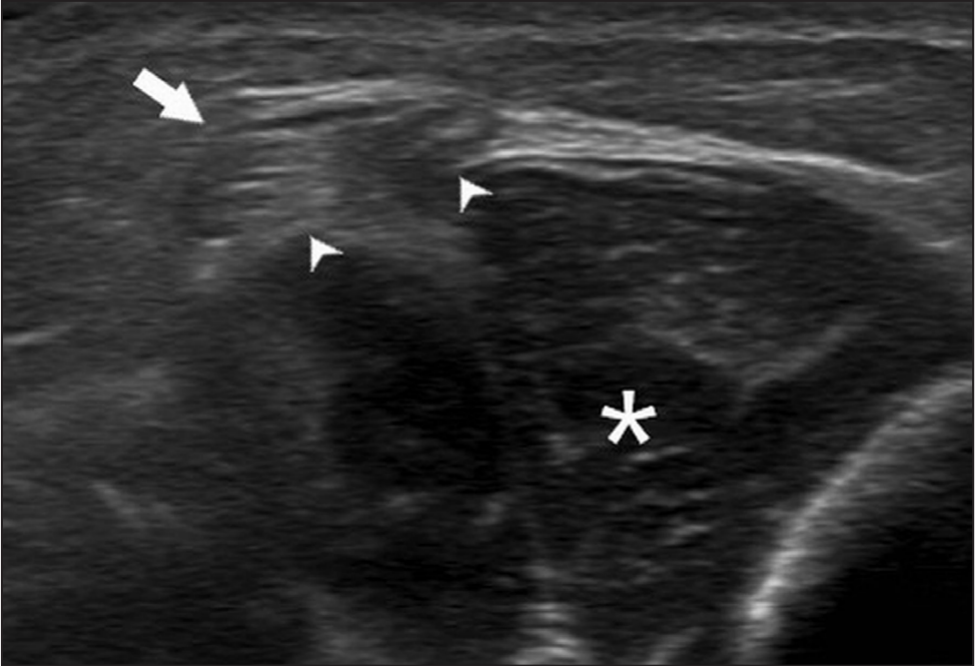


**Figure 2:**
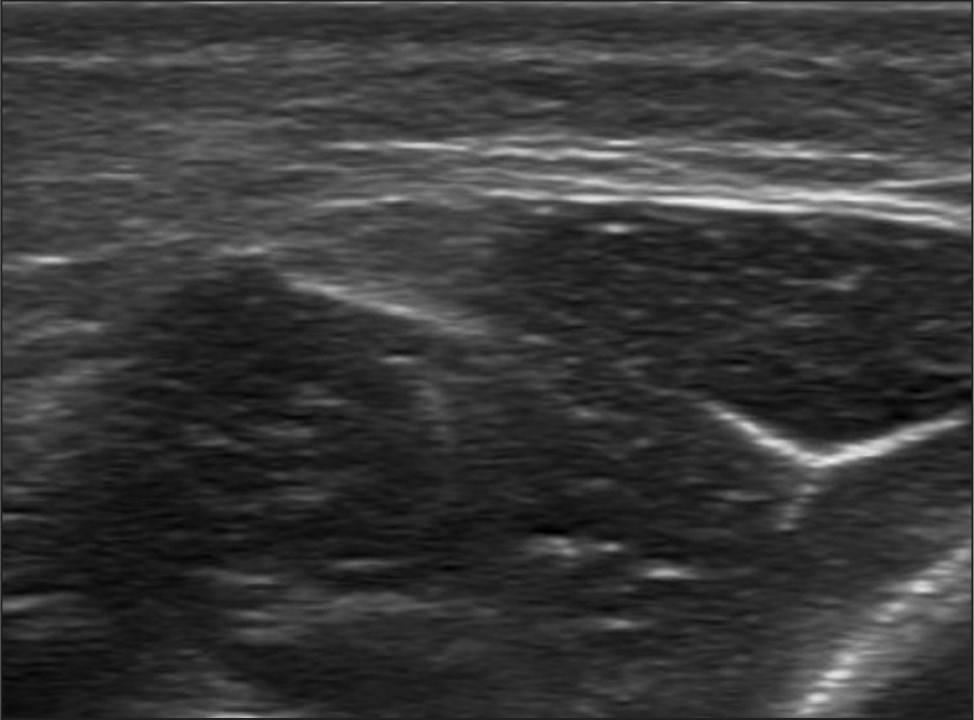


**Figure 3:**
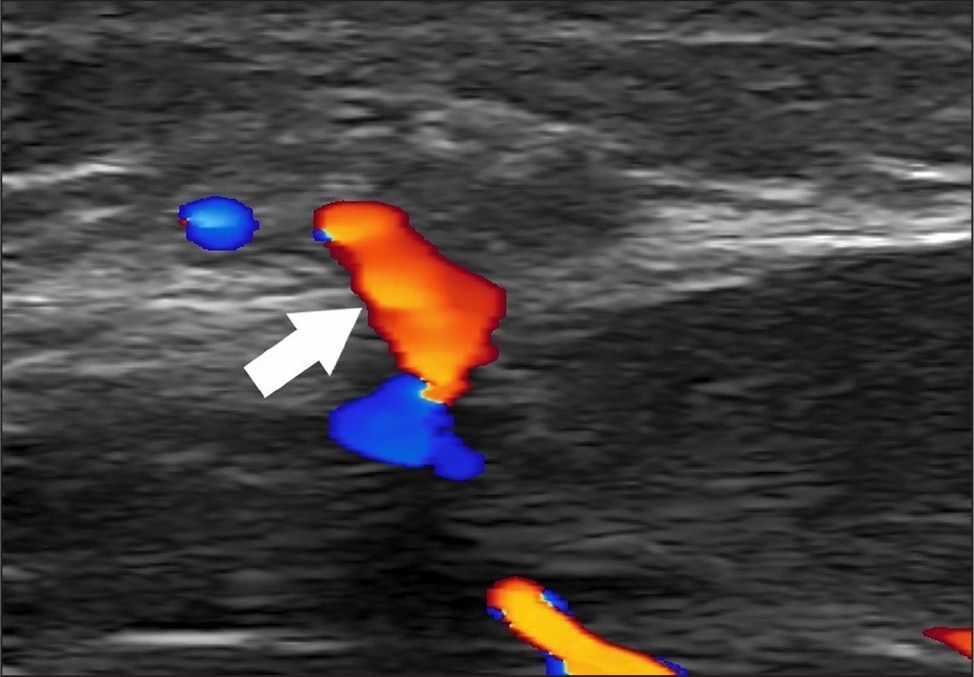


A one-year-old girl presented a less-than-one centimeter palpable subcutaneous nodule of the anterior middle third of the left leg, noticed by her mother few months after delivery. No traumatic circumstances were reported by parents. Ultrasonography showed a defect of 3 mm in the fascia (*arrowheads*) of the tibialis anterior (*asterisk*) with an outward bulge of the outer fibers of the muscle through the defect in the subcutaneous tissues (*arrow*). The muscle hernia appeared heterogeneous, as a hypoechoic mass with hyperechoic septa. The hernia presented a convex contour, getting a mushroom-like appearance (Figure 1). The herniation was partially reducible with the transducer compression (Figure 2). Small vessels (*arrow*) were seen in the fascial defect, adjacent to the muscle herniation (Figure 3).

## Comment

Muscle hernias are acquired (post-traumatic) or congenital (caused by a general weakness of muscular fascia or occur at sites of focal fascial weakness). They usually occur in the lower extremities. The most common site is the anterolateral compartment of the leg, where the fascia is superficial and tight. The tibialis anterior is the most frequently involved muscle. Muscle hernia presents as a soft tissue mass or subcutaneous nodule that may vary with muscle contraction. It is usually reducible, but can be irreducible when the muscle is strangulated in the hernia. Most of the time, muscle hernias are asymptomatic but patients may have pain, tenderness, weakness, cramping or decreased sensation.

The main differential diagnoses are angiomas, arteriovenous malformation, lipomas, ruptured muscle and soft tissue tumors.

Muscle hernias can be diagnosed by ultrasonography or magnetic resonance imaging (MRI). Ultrasonography should be performed with the patient in the best position to reveal the hernia. Provocative maneuvers can be useful. A spoke-like appearance is sometimes described and is related to normal echoic fibroadipose septa protruding through and radiating from the center of the fascial defect. Sometimes, Doppler imaging shows vessels at the site of herniation. This observation supports the theory that hernias can occur at areas of fascial weakness, such as the entrance sites of perforating vessels and should not be confused with a sign in favor of a tumor mass.

MRI may show a better visualization of defect, despite the fact that some cases missed by MRI have been reported in literature, probably due to a too small size of fascial interruption. Subtle hyperintense signal on T2-weighted sequence suggestive of muscle edema can be seen [1].

In conclusion, muscle hernias are not rare, but most of them may stay undiagnosed due to their asymptomatic presentation. Acquired cases are usually described, but congenital form is acknowledged. This case confirms the existence of congenital hernia in very young pediatric population suggesting a possibility of perinatal fascia interruption. Although rarely diagnosed as early, muscle hernias should be considered in the differential diagnosis of infantile leg masses.
